# Somatic Symptoms: Prevalence, Co-Occurrence and Associations with Self-Perceived Health and Limitations Due To Physical Health – A Danish Population-Based Study

**DOI:** 10.1371/journal.pone.0150664

**Published:** 2016-03-01

**Authors:** Marie Eliasen, Svend Kreiner, Jeanette F. Ebstrup, Chalotte H. Poulsen, Cathrine J. Lau, Sine Skovbjerg, Per K. Fink, Torben Jørgensen

**Affiliations:** 1 Research Centre for Prevention and Health, The Capital Region of Denmark, Glostrup, Denmark; 2 Department of Public Health, University of Copenhagen, Copenhagen, Denmark; 3 Mental Health Centre Copenhagen, The Capital Region of Denmark, Gentofte, Denmark; 4 Research Clinic for Functional Disorders and Psychosomatics, Aarhus University Hospital, Aarhus, Denmark; 5 Department of Clinical Medicine, Aalborg University, Aalborg, Denmark; S.G.Battista Hospital, ITALY

## Abstract

A high number of somatic symptoms have been associated with poor health status and increased health care use. Previous studies focused on number of symptoms without considering the specific symptoms. The aim of the study was to investigate 1) the prevalence of 19 somatic symptoms, 2) the associations between the symptoms, and 3) the associations between the somatic symptoms, self-perceived health and limitations due to physical health accounting for the co-occurrence of symptoms. Information on 19 somatic symptoms, self-perceived health and limitations due to physical health was achieved from a population-based questionnaire survey of 36,163 randomly selected adults in the Capital Region of Denmark in 2006/07. Chain graph models were used to transparently identify and describe the associations between symptoms, self-perceived health and limitations due to physical health. In total, 94.9% of the respondents were bothered by one or more of the 19 somatic symptoms. The symptoms were associated in a complex structure. Still, recognisable patterns were identified within organ systems/body parts. When accounting for symptom co-occurrence; dizziness, pain in legs, respiratory distress and tiredness were all strongly directly associated with both of the outcomes (γ>0.30). Chest pain was strongly associated with self-perceived health, and other musculoskeletal symptoms and urinary retention were strongly associated with limitations due to physical health. Other symptoms were either moderate or not statistically associated with the health status outcomes. Opposite, almost all the symptoms were strongly associated with the two outcomes when not accounting for symptom co-occurrence. In conclusion, we found that somatic symptoms were frequent and associated in a complex structure. The associations between symptoms and health status measures differed between the symptoms and depended on the co-occurrence of symptoms. This indicates an importance of considering both the specific symptoms and symptom co-occurrence in further symptom research instead of merely counting symptoms.

## Introduction

The experience of somatic symptoms such as headache, musculoskeletal pain and tiredness is common in the general population [1–5]. A high number of somatic symptoms has been associated with increased health care use [6], poor health status [6], poor functional status [7,8], and sickness absence [4,9] independently of the aetiology of the symptoms. Thus, whether medically explained or not, somatic symptoms are an important aspect of the health status. Still, focus in health research has mostly been on diseases/disorders or on a few specific symptoms instead of focusing on multiple somatic symptoms in general [5].

In the existing research on multiple symptoms and their association with health outcomes, focus has mostly been on number of symptoms without considering the specific symptoms. However, when counting symptoms, it is assumed that all symptoms count evenly in relation to the outcome despite that it is likely that some symptoms will predict worse outcomes than others. Merely counting symptoms can therefore distort the associations between symptoms and health status measures or at least conceal some useful information. Bruusgaard et al. investigated the association between symptoms and functional status using information on both specific symptoms and symptom count [7]. They found that the associations differed according to the specific symptoms and depended on the adjustment for other symptoms. Moreover, they showed that the specific symptoms explained more of the variance in functional status than did the number of symptoms. Still, the number of symptoms explained a substantial part of the variance [7]. Hence, these results emphasise that both the specific symptoms and the co-occurrence of symptoms should be accounted for in symptom research instead of merely counting the symptoms or looking at a few specific symptoms [5,10]. However, the study had some limitations including differences between the measurement on musculoskeletal and non-musculoskeletal symptoms [7]. Furthermore, they applied standard regression models which cannot account for the pattern of symptom co-occurrence. The co-occurrence of symptoms can be understood as a system in which the symptoms affect each other in different patterns. Investigating and accounting for this symptom system can give a better insight of the co-occurrence of somatic symptoms and their association with health status measures as can the use of different measures of the health status. This insight can then help in guiding further epidemiological somatic symptom research.

In this study, we had the following three aims: 1) to describe the prevalence of common somatic symptoms in the general adult population, 2) to describe the system in which the symptoms co-occurred using chain graph models, and 3) to investigate the associations between multiple somatic symptoms, self-perceived health and limitations due to physical health accounting for the system of co-occurring symptoms.

## Methods

### Study population

We used data from a cross-sectional health and life-style survey “The Regional Health Survey 2006/2007” conducted in the Capital Region of Denmark in the summer–autumn 2006 and 2007. Municipality-stratified random samples of the general population aged 25–79 years old were drawn from the Danish Civil Registration System—each citizen in Denmark has a unique personal registration number—using computer generated random numbers [11,12]. The selected individuals (N = 69,800) were sent an invitation to complete an enclosed questionnaire. A postal reminder was sent containing a new questionnaire. Of those invited, 36,472 (52%) returned a completed questionnaire.

The survey study including the consent procedure was approved by the Danish Data Protection Board, while the study did not require approval from an ethics committee. Written informed consent was given by the participants by returning the questionnaire.

### Somatic symptoms

Information on somatic symptoms was assessed by a question on how much the participant had been bothered by 19 listed somatic symptoms 14 days prior to answering the questionnaire. Possible answers were “not at all”, “a little” and “a lot”. The list covered the most frequent symptoms reported in other studies [13] and included three musculoskeletal symptoms, three cardiopulmonary symptoms, three gastrointestinal symptoms, two urinary tract symptoms and eight other symptoms (Table 1). The list also included a 20^th^ somatic symptom: “lower abdominal pain or intense menstrual pain”, but as it was seen as a highly sex specific symptom, we omitted it in the analyses.

**Table 1:** The prevalence of 19 experienced somatic symptoms during 14 days including the proportion of women and the median age in the three symptom categories. The Regional Health Survey 2006/2007, the Capital Region of Denmark. N = 35,122–35,810.

	Prevalence in total population (%)			Proportion of women (%)			Median age (years)		
Symptom	Bothered in total	Somewhat bothered	Considerably bothered	Not bothered	Somewhat bothered	Considerably bothered	Not bothered	Somewhat bothered	Considerably bothered
Tiredness	60.7	48.8	11.9	44.0	53.7	62.0	49.7	43.9	43.8
Neck or shoulder pain	50.6	38.6	12.0	43.4	56.1	64.7	44.4	46.6	49.4
Back pain	49.7	37.5	12.2	47.7	52.0	59.6	44.0	47.2	50.3
Pain in leg/hip/knee	44.2	32.4	11.8	49.4	50.8	57.8	41.8	51.2	55.2
Headache	41.2	34.4	6.8	43.6	59.0	70.9	50.2	41.5	41.9
Cold, running nose, coughing	33.6	27.2	6.4	51.3	47.6	56.6	47.2	43.2	43.0
Sleeplessness	33.5	26.9	6.6	48.0	56.1	59.7	44.2	49.1	50.2
Stomach pain/ abdominal distension	32.5	27.2	5.3	46.4	58.5	68.1	47.0	43.7	45.5
Indigestion, loose/hard stools	27.8	23.0	4.8	48.7	54.4	65.1	46.0	45.7	47.8
Skin rash, itching, eczema	21.3	17.6	3.7	51.0	48.9	56.5	45.7	47.6	46.8
Respiratory distress	20.6	17.1	3.5	51.2	49.5	49.0	44.1	53.0	57.7
Dizziness	17.9	15.4	2.5	48.7	59.8	58.8	45.3	48.1	51.4
Impaired hearing	17.7	14.8	2.9	52.8	42.0	43.4	43.5	58.5	61.8
Impaired vision	17.0	14.6	2.4	50.4	52.9	57.2	43.8	54.8	56.0
Rapid heart beat	14.6	13.0	1.6	49.7	57.8	57.4	45.5	48.1	49.6
Urinary incontinence	12.7	10.5	2.2	48.6	68.5	65.9	44.2	58.4	59.7
Nausea	11.8	10.2	1.6	48.8	65.5	70.5	46.5	42.2	44.3
Chest pain/discomfort	11.1	9.8	1.3	51.3	47.1	51.8	45.5	49.8	51.3
Urinary retention	4.1	3.4	0.7	52.1	23.3	30.0	45.2	62.1	60.6

All numbers are weighted to account for sampling procedure and non-response. The symptoms are sorted after prevalence. The size of the study population differs according to missing on symptoms.

Column explanation: “Prevalence in total population (%)”: The percentage of the population bothered by the symptoms; totally and divided into somewhat and considerably bothered. “Proportion of women (%)”: the proportion of women in the three symptom categories. “Median age (years)”: The median age in the three symptom categories.

### Health status measures

Self-perceived health was measured by the question “In general, would you say your health is:” with answers on a 5-point scale from excellent to poor. We combined the categories into the following: “excellent/very good”, “good” and “fair/poor”.

Limitations due to physical health were assessed by a single question asking the participants to state if their physical health had limited them in their work abilities or in other activities in a 4-week period preceding the survey. The five possible responses were collapsed into three categories: 1) “considerably limited” (combination of “all of the time”, “most of the time” and “some of the time”), 2) “somewhat limited” (“a little of the time”) and 3) “not limited” (“none of the time”).

### Covariates

Information on age and sex was extracted from the personal registration number. Age was categorised into three groups: <40 years, 40–60 years and >60 years.

### Final study population

The size of the study population differed in the analyses using most information available. Information was missing on all the symptoms for 309 individuals leaving a maximum of 36,163 for the analyses. Information on self-perceived health and limitations due to physical health was missing on 393 and 901 individuals leaving a maximum of 35,770 and 35,626 for these analyses, respectively.

Full information on all symptoms was available for 32,508 (89.9%) individuals, while full information on all the symptoms and the two health status measures was available for 32,199 (89.0%) and 31,953 (88.4%), respectively.

### Statistical methods

The prevalence of the 19 somatic symptoms was calculated in SAS software (SAS Institute Inc., Cary, NC, USA) using weights to account for sampling procedure and non-response (Table 1). The weighting for non-response was based on information from Statistics Denmark on sex, age, civil status, ethnicity, education, income, working conditions, hospital admissions and medical visits [11,14].

To assess the system of symptom co-occurrence and the associations between symptoms, self-perceived health and limitations due to physical health, we applied log-linear chain graph models [15] which can be regarded as generalizations of the causal models defined by Directed Acyclic Graphs (DAGs) [16–18]. A chain graph model constitutes of nodes representing variables, directed arrows representing causal associations and, opposite to DAGs, undirected edges representing non-causal associations. Chain graph models have a block structure with arrows between blocks and edges within blocks. Using the graph structure, all paths between two variables can be determined; hereby, it is possible to identify a minimum set of variables to condition on when estimating the direct association between two variables of interest. For further discussion of analysis by graphical models see [15,19–21]. By using chain graph models to assess the co-occurrence of symptoms, we could obtain and illustrate the correlation structure of the symptoms graphically based on observed data instead of either a predefined structure or inclusion of all symptoms into a regression model, which does not account for the structure.

**Fig 1:**
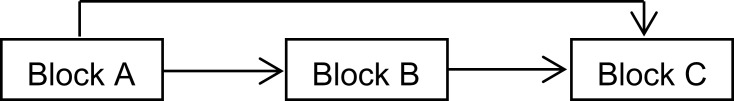
Model of the block structure of the chain graph model used in the study. Block A: age and sex. Block B: the 19 somatic symptoms in a interrelated system. Block C: self-perceived health or limitations due to physical health.

Fig 1 shows the block structure underlying the analysis. Block A consisted of age and sex with arrows pointing towards the other blocks. Block B consisted of all the symptoms without assumptions of causal associations between the variables. Finally, block C consisted of self-perceived health or limitations due to physical health.

To determine the correlation structure based on statistically significant correlations and to estimate the size of the correlations, we used partial γ (gamma) coefficients [22,23], which are rank correlation coefficients for ordinal categorical data, where a γ-value>0.30 was regarded as evidence of a strong association. We defined and tested the chain graph model in DIGRAM [24]. We firstly defined an initial model using an extended strategy for screening of high-dimensional contingency tables [25] and then used a stepwise non-automatic procedure aimed at identifying an adequate model for data. The non-automatic procedure was based on the strength of the associations, the p-values and our clinical knowledge of the associations. Associations between symptoms with γ-values below 0.10 were deleted from the model as these indicated very weak associations. We accounted for multiple tests using the Benjamini-Hochberg procedure [26], while asymptotic problems in estimating the γ-coefficients and p-values were accounted for using the Monte Carlo procedure [23].

Based on the identified model, we estimated the partial correlations between symptoms and the health status measures conditioning on the minimum set of necessary symptoms, age and sex (Table 2, Model B). Statistical interactions between age, sex and the symptoms according to the health status measures were investigated simultaneously. To explore the impact of including information on co-occurring symptoms, we also estimated the associations between symptoms, self-perceived health and limitations due to physical health adjusting only for age and sex (Table 2, Model A).

**Table 2:** Associations between 19 experienced somatic symptoms, self-perceived health and limitations due to physical health. N = 32,762–33,010.

	Self-perceived health				Limitations due to physical health			
		Model A^a^		Model B^b^		Model A^a^		Model B^b^
Symptom	γ	(95% CI)	γ	(95% CI)	γ	(95% CI)	γ	(95% CI)
Tiredness	0.62	(0.61–0.63)	0.37*	(0.30–0.44)	0.56	(0.55–0.58)	0.38*	(0.29–0.48)
Neck or shoulder pain	0.50	(0.48–0.51)	0.22*	(0.15–0.29)	0.52	(0.50–0.53)	0.36*	(0.27–0.44)
Back pain	0.51	(0.50–0.52)	0.23*	(0.16–0.29)	0.55	(0.54–0.57)	0.47*	(0.39–0.56)
Pain in leg/hip/knee	0.56	(0.54–0.57)	0.37*	(0.30–0.44)	0.61	(0.60–0.63)	0.65*	(0.56–0.74)
Headache	0.36	(0.34–0.37)	-0.02	(-0.10–0.06)	0.32	(0.30–0.34)	0.13*	(0.03–0.22)
Cold, running nose, coughing	0.25	(0.23–0.27)	0.11*	(0.03–0.18)	0.21	(0.19–0.24)	0.20*	(0.09–0.30)
Sleeplessness	0.44	(0.43–0.46)	0.22*	(0.13–0.30)	0.38	(0.36–0.40)	0.17*	(0.06–0.27)
Stomach pain/ abdominal distension	0.39	(0.38–0.41)	0.14*	(0.05–0.22)	0.32	(0.30–0.34)	-0.09	(-0.20–0.02)
Indigestion, loose/hard stools	0.42	(0.40–0.44)	0.13	(0.00–0.65)	0.37	(0.35–0.39)	0.22*	(0.09–0.36)
Skin rash, itching, eczema	0.30	(0.27–0.31)	0.05	(-0.06–0.15)	0.25	(0.22–0.27)	0.06	(-0.08–0.19)
Respiratory distress	0.65	(0.63–0.66)	0.55*	(0.41–0.68)	0.59	(0.57–0.61)	0.59*	(0.44–0.27)
Dizziness	0.59	(0.58–0.61)	0.30*	(0.13–0.47)	0.55	(0.53–0.57)	0.38*	(0.16–0.61)
Impaired hearing	0.31	(0.29–0.33)	0.17*	(0.06–0.28)	0.29	(0.27–0.31)	0.15	(0.00–0.29)
Impaired vision	0.44	(0.42–0.46)	0.25*	(0.11–0.40)	0.41	(0.39–0.44)	0.27*	(0.11–0.42)
Rapid heart beat	0.57	(0.55–0.59)	0.14	(-0.05–0.32)	0.49	(0.47–0.52)	0.16	(0.04–0.36)
Urinary incontinence	0.40	(0.37–0.42)	0.25*	(0.10–0.43)	0.40	(0.38–0.43)	0.21	(0.04–0.38)
Nausea	0.56	(0.53–0.58)	0.24*	(0.05–0.43)	0.54	(0.52–0.57)	0.29*	(0.04–0.54)
Chest pain/discomfort	0.60	(0.57–0.62)	0.36*	(0.15–0.58)	0.52	(0.49–0.54)	0.09	(-0.13–0.32)
Urinary retention	0.52	(0.48–0.55)	0.32	(0.00–0.65)	0.48	(0.45–0.52)	0.40*	(0.07–0.72)

γ: Partial correlation coefficients. A positive γ-value indicates that the symptom is associated with an increased chance of poor self-perceived health and having limitations due to physical health. A γ-value>0.30 can be regarded as a strong association. CI: Confidence interval. The size of the study population differs according to missing on symptoms.

^a^ Model A: Adjusted for age and sex.

**Fig 2:**
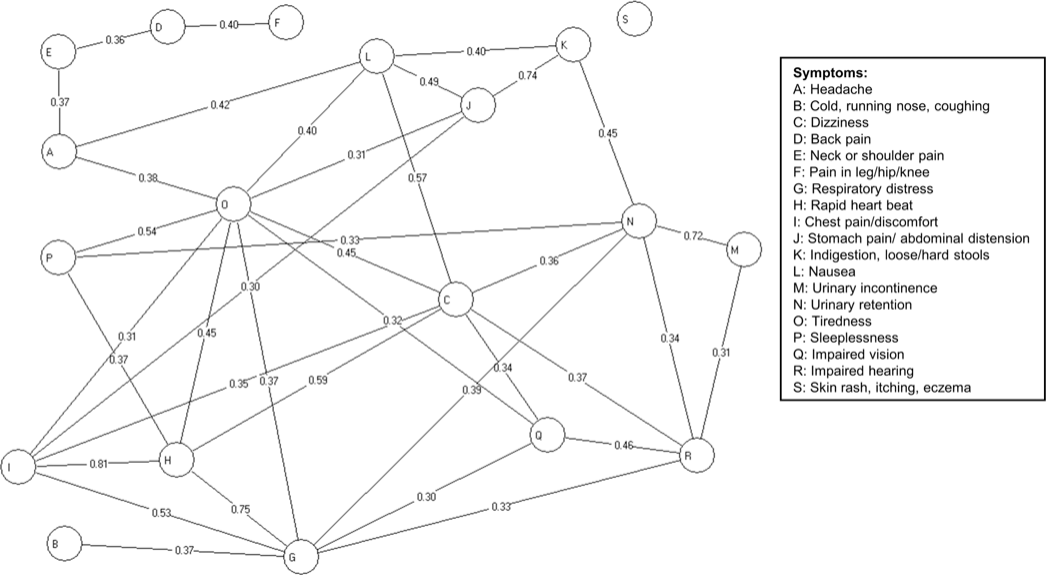
Chain graph model showing the associations between symptoms. All lines represent significant conditional associations. The numbers indicate the partial γ-coefficients. To reduce the complexity of the figure, only strong associations, i.e. γ>0.30, are shown, while associations with 0.10<γ<0.30 are included in the statistical model but not in the figure. The associations are adjusted for age and sex (not shown in the figure).

^b^ Model B: Adjusted for co-occurrence of symptoms, sex and age according to the chain graph chain model in Fig 2.

* Statistically significant at a 0.01 level when accounting for multiple testing using the Benjamini-Hochberg procedure.

To evaluate the impact of missing on symptoms, we conducted two sensitivity analyses: 1) treating missing values as “not bothered” assuming that the participants would have answered if they were bothered by the symptom, and 2) excluding all persons with missing values.

## Results

### Characteristics of the population and prevalence of symptoms

The 36,163 persons included in the study had a median age of 46.2 years (10^th^ and 90^th^ percentile: 29.0–68.5 years) and 51.0% were women. In total, 94.9% of the persons had been at least somewhat bothered by one or more symptoms during the 14 days preceding the survey, while 39.6% of the persons had been considerably bothered by one or more symptoms. The percentages for three or more symptoms were 75.7% for being at least somewhat bothered and 13.7% for being considerably bothered.

Table 1 shows the weighted prevalence of the 19 somatic symptoms, the proportion of women and the median age in the three symptom categories. The most common symptoms were tiredness affecting 60.7% of the population and musculoskeletal symptoms (back pain, neck/shoulder pain and pain in leg/hip/knee) each with a prevalence of around 50% (Table 1). Being considerably bothered by these symptoms was reported by 11.8–12.2% of the population. Headache was also common with a prevalence of 41.2%, but the far majority had only been somewhat bothered by it. For most of the symptoms, a higher proportion of women and a higher median age were associated with being considerably bothered by the symptom (Table 1). Exceptions were urinary retention and impaired hearing with a higher fraction of men being bothered, and breathing difficulties and chest pain that were not associated with sex. Exceptions for age included headache, cold and tiredness, which were associated with a younger median age, and gastrointestinal symptoms and skin rash which were not associated with age.

### Co-occurrence and associations between the symptoms

Fig 2 shows all the strong associations between the symptoms (γ>0.30). Generally, the strongest associations were found between symptoms from the same body part or organ system. Thus, based on their correlations, the symptoms could overall be categorised in the following groups: 1) musculoskeletal pain (back pain, neck/shoulder pain and pain in leg/hip/knee), 2) gastrointestinal symptoms (stomach pain/abdominal distension and indigestion), 3) cardiopulmonary symptoms (respiratory distress, rapid heartbeat and chest pain), 4) urinary tract symptoms (urinary incontinence and retention), and 5) symptoms from the central nervous system and more general symptoms (tiredness, sleeplessness, dizziness, headache and nausea). However, the symptoms were also highly correlated across the five groups. Especially, the general symptoms and respiratory distress showed strong correlations with symptoms across the groups. When looking at the low and moderate size associations, even more symptoms were correlated across groups in a complex structure (results not shown). Only cold and skin rash showed low (γ<0.23) or no correlation with the other symptoms, except for the correlation between cold and breathing difficulties (γ = 0.37).

### Somatic symptoms and self-perceived health

When looking at the somatic symptoms separately, all were significantly positively associated with poor self-perceived health (Table 2, Model A). Thus, the more bothered by the symptoms, the worse self-perceived health. All the correlations were moderate to strong, but did still differ in size (0.25≤γ≤0.65). When accounting for symptom co-occurrence, all the correlation coefficients attenuated, and headache, rapid heartbeat, indigestion, urinary retention and skin rash were no longer significantly associated with self-perceived health (Table 2, Model B; S1A Appendix). Dizziness, pain in the leg/hip/knee, respiratory distress, chest pain and tiredness were still strongly correlated with self-perceived health (γ>0.30), and especially respiratory distress showed a high correlation coefficient (γ = 0.55). The remaining symptoms showed low to moderate associations with self-perceived health when accounting for symptom co-occurrence.

Age and sex did not interact statistically with any of the symptoms in their association with self-perceived health (results not shown).

### Somatic symptoms and limitations due to physical health

All the symptoms were significantly positively associated with limitations due to physical health when looking at the symptoms separately, showing moderate to strong correlations (Table 2, Model A). When accounting for symptom co-occurrence; rapid heartbeat, chest pain, stomach pain, urinary incontinence, impaired hearing and skin rash were no longer statistically significantly associated with limitations due to physical health. Dizziness, musculoskeletal symptoms, respiratory distress, urinary retention and tiredness were still strongly correlated with the outcome (γ>0.30), however the correlation coefficient for urinary retention had very wide confidence limits (95% CI: 0.07–0.72) (Table 2, Model B; S1B Appendix). Back pain, pain in the legs and respiratory distress showed very strong correlations (γ between 0.48 and 0.64).

Respiratory distress and tiredness were significantly stronger associated with limitations due to physical health among older people than younger. Thus, for respiratory distress the γ-values were 0.60 (95% CI: 0.44–0.76) for 40+ year-olds and 0.22 (-0.02–0.46) for younger. For tiredness, the γ-values were 0.57 (0.41–0.73) for 60+ year-olds and 0.25 (0.14–0.36) for younger. Chest pain also interacted with age having a negative correlation among <40 year-olds (γ = -0.44; 95% CI: -0.66–-0.22), and no association among older (γ = 0.13; 95% CI: -0.07–0.33). This interaction could however not be found in the analysis adjusting for only age and sex.

The analyses excluding all with missing values and treating missing values as not bothered did not differ noteworthy from the main analyses with a maximum difference on +/-0.03 in the correlation coefficients (results not shown).

## Discussion

In this large population-based study, we found a high frequency of somatic symptoms with tiredness and musculoskeletal symptoms as the most prevalent. The symptoms correlated with each other in a complex structure with some recognisable patterns within organ systems/body parts. When accounting for symptom co-occurrence; dizziness, pain in legs, respiratory distress and tiredness correlated strongly with both self-perceived health and limitations due to physical health. Chest pain correlated strongly with self-perceived health, while other musculoskeletal symptoms and urinary retention correlated strongly with limitations due to physical health. When looking at the symptoms separately, almost all the symptoms correlated strongly with the two health outcomes.

### Relation to other studies

To our knowledge, no previous studies have looked at symptom co-occurrence of multiple symptoms in detail by showing the associations and patterns graphically ensuring a transparent description of the symptom system. However, the pattern of symptom co-occurrence has previously been studied using factor analyses [27–35]. Different numbers and types of factors were identified, but often a musculoskeletal pain, a cardiopulmonary, and a gastrointestinal factor was found [13,27–29,34,35]. This is in accordance with our findings of strong correlations between symptoms in these categories. Some of the studies also found a general factor with loadings from all symptoms [30,35]; this could correspond to our findings of correlations between symptoms across organ systems and body parts.

Several studies have investigated the association between symptoms and different health status measures (e.g. [6,7,10,36,37]). Mostly, the studies have focused on either number of symptoms or single symptoms. The studies showed that a high number of symptoms or a specific single symptom were associated with poor health status. However, to our knowledge, only the study by Bruusgaard et al. had investigated the impact of multiple specific symptoms accounting for other symptoms using physical functioning as the health status outcome [7]. They found that breathing difficulties and pain in the upper and lower back, hips and knees had the highest associations with physical functioning when controlling for other symptoms, which is partly in accordance with our findings of symptoms and limitations due to physical health. However, tiredness and dizziness had only low associations with physical functioning opposite to what we found in our study. Bruusgaard et al. also found that the associations between symptoms and physical functioning attenuated when controlling for the other symptoms similar to our findings [7].

The findings of a high prevalence of somatic symptoms and the commonness of tiredness and musculoskeletal symptoms confirm findings from previous studies [1–5,8,9,27,38]. However, the exact prevalence estimates of the specific symptoms differ between the studies. This might partly be explained by the time window of symptom measurement and measurements of either severity or frequency of symptoms [13]. Moreover, the number and type of symptoms measured make comparisons difficult [13]. Especially, impaired hearing and vision might not typically be included in somatic symptom research [13]. However, the prevalence estimate for being bothered by any symptoms did not change notably when excluding the two symptoms (94.3% vs. 94.9%). We included the symptoms to use as much information as possible.

### Methodological considerations

Strengths of this study included the investigation of a variety of somatic symptoms in a population-based sample covering the most frequently reported symptoms in other studies [13,27]. The symptoms were all measured using the same question and the same time window. We used chain graph models to explore the system of symptom co-occurrence and to account for this co-occurrence when investigating the associations between symptoms and health status measures. This method enhanced the transparency of the symptom structure and ensured an adjustment of the associations using a minimum set of symptoms. This is opposite to a standard regression model in which all symptoms would be mutually adjusted for each other, and the structure of the model would be implicit reducing the transparency. Another advantage of using the chain graph model is the use of partial γ-coefficients accounting for the ordered structure of both symptom severity and health status measures.

Still, the model had some limitations. We predefined the causal structure in the model, but this could not be tested and might not be equivalent to real life situations. Thus, the model should only be interpreted as a theoretical model used to analyse the data. Moreover, the associations should only be interpreted as predictions and not as causal, as several factors might explain some of the association between the symptoms and health status measures, e.g. well-defined disease, socio-economic position and personality. However, we did not account for any confounders except age and sex as we wished to 1) investigate how somatic symptoms predicted health status irrespective of the cause of the symptoms, which could be used in further research to identify vulnerable groups based on symptom presentation, 2) to illustrate the importance of differing between symptoms while considering symptom co-occurrence to guide further research, and 3) to describe symptom co-occurrence transparently without considering explaining factors to increase the knowledge of somatic symptom co-occurrence irrespective of aetiology.

Another potential limitation with the results is the relatively low response rate which may bias the results and affect the generalisability. Participants were in general older, had longer education, higher income and were more likely to live together with other people compared with non-participants [12], which could indicate healthier participants than non-participants. This could result in an underestimation of the symptom prevalence. We tried to account for this by weighting the prevalence estimates according to socio-demographic factors. The selection process could also have biased the associations between symptoms and between symptoms and health status measures which would most likely be towards the null. Furthermore, missing on symptom reporting could also have biased the results, but as the associations did not change notably when excluding all participants with minimum one missing or when assuming missing was equal to not having the symptom, this possible bias was assumed to be of minor importance. Moreover, if the selection and missing affected the associations the same way, the comparisons and tendencies of correlation sizes would be approximately the same and hence generalizable to other populations.

### Conclusion and perspectives

In this study, we found that somatic symptoms were frequent in the general population and were correlated in a complex structure but still with some recognisable patterns. When accounting for symptom co-occurrence, several symptoms were highly correlated with self-perceived health and/or limitations due to physical health while other symptoms were not. The symptoms should be seen as predictors of poor health status and not as causal factors due to possible confounding, and the symptom system should be seen as an indicator of which symptoms often co-occur. The differences in correlation sizes could likely be generalised and should therefore be considered in further somatic symptom research. Thus, we argue that both the single symptoms and the co-occurrence of symptoms are important instead of merely assessing the number of symptoms or looking at a few single symptoms. Together with the findings of the complex structure of symptom co-occurrence, the study may further indicate a need for a new way of identifying persons with poor prognosis and poor health status based on their symptom reporting especially to be used in further epidemiological research.

## Supporting Information

S1 Appendix Chain graph models of the association between symptoms and A) self-perceived health and B) limitations due to physical health. All lines represent significant conditional associations. The numbers indicate the partial γ-coefficients. To reduce the complexity of the figure, only strong associations, i.e. γ>0.30, are shown, while associations with 0.10<γ<0.30 are included in the statistical model but not in the figure. The associations are adjusted for age and sex (not shown in the figure). Dark grey nodes represent significant and strong associations with the outcome (γ>0.30, p<0.01). Light grey nodes represent significant and weak to moderate associations (0.10<γ<0.30, p<0.01). White nodes represent non-significant associations (p>0.01).
(DOCX)
