# Supplementary material for: Somatic Symptoms: Prevalence, Co-Occurrence and Associations with Self-Perceived Health and Limitations Due To Physical Health – A Danish Population-Based Study
Source: PLoS One. 2016 Mar 1;11(3):e0150664. doi: 10.1371/journal.pone.0150664 (PMC4773248; doi:10.1371/journal.pone.0150664)
Supplement: S1 Appendix — Chain graph models of the association between symptoms and A) self-perceived health and B) limitations due to physical health. All lines represent significant conditional associations. The numbers indicate the partial γ-coefficients. To reduce the complexity of the figure, only strong associations, i.e. γ>0.30, are shown, while associations with 0.10<γ<0.30 are included in the statistical model but not in the figure. The associations are adjusted for age and sex (not shown in the figure). Dark grey nodes represent significant and strong associations with the outcome (γ>0.30, p<0.01). Light grey nodes represent significant and weak to moderate associations (0.10<γ<0.30, p<0.01). White nodes represent non-significant associations (p>0.01). (DOCX) [file pone.0150664.s001.docx]

**S1 Appendix. Chain graph models of the association between symptoms and A) self-perceived health and B) limitations due to physical health**. All lines represent significant conditional associations. The numbers indicate the γ-coefficients. To reduce the complexity of the figure, only strong associations, i.e. γ>0.30, are shown, while associations with 0.10<γ<0.30 are included in the statistical model but not in the figure. The associations are adjusted for age and sex (not shown in the figure). Dark grey nodes represent significant and strong associations with the outcome (γ>0.30, p<0.01). Light grey nodes represent significant and weak to moderate associations (0.10<γ<0.30, p<0.01). White nodes represent non-significant associations (p>0.01).

**A) Somatic symptoms and self-perceived health (SPH)**

**
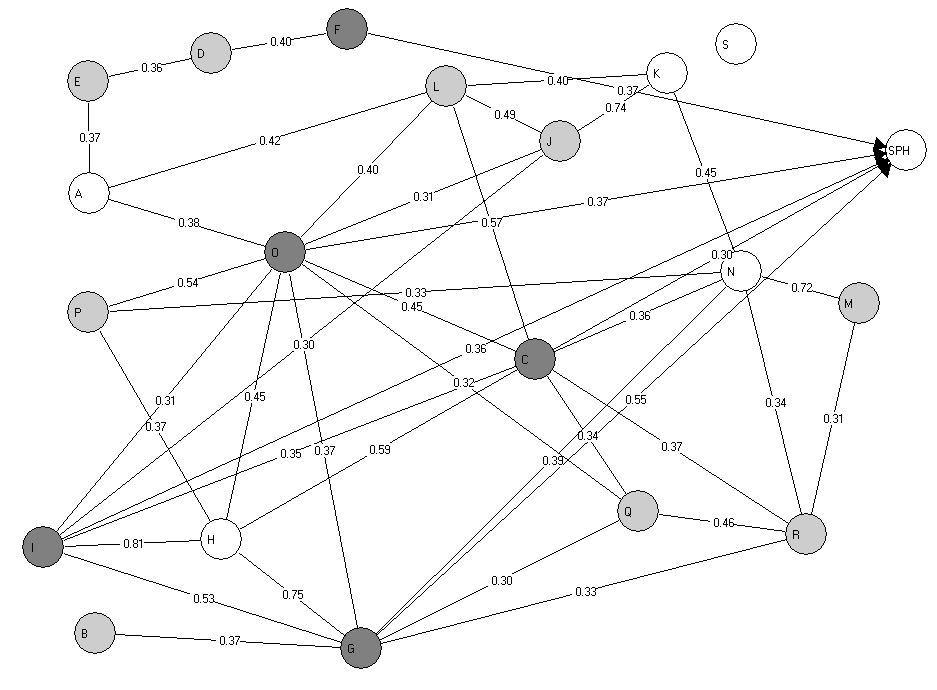
**

**Symptoms:**

A: Headache

B: Cold, running nose, coughing

C: Dizziness

D: Back pain

E: Neck or shoulder pain

F: Pain in leg/hip/knee

G: Respiratory distress

H: Rapid heart beat

I: Chest pain/discomfort

J: Stomach pain/ abdominal distension

K: Indigestion, loose/hard stools

L: Nausea

M: Urinary incontinence

N: Urinary retention

O: Tiredness

P: Sleeplessness

Q: Impaired vision

R: Impaired hearing

S: Skin rash, itching, eczema

**S1 Appendix (continued)**

**B) Somatic symptoms and limitations due to physical health (limits)**

**
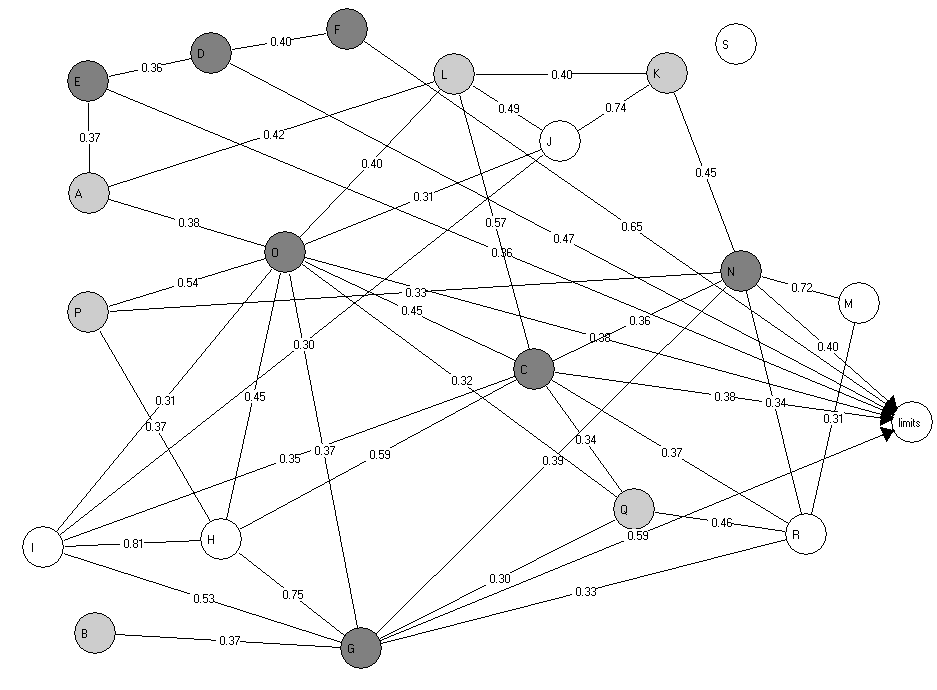
**

**Symptoms:**

A: Headache

B: Cold, running nose, coughing

C: Dizziness

D: Back pain

E: Neck or shoulder pain

F: Pain in leg/hip/knee

G: Respiratory distress

H: Rapid heart beat

I: Chest pain/discomfort

J: Stomach pain/ abdominal distension

K: Indigestion, loose/hard stools

L: Nausea

M: Urinary incontinence

N: Urinary retention

O: Tiredness

P: Sleeplessness

Q: Impaired vision

R: Impaired hearing

S: Skin rash, itching, eczema
